# A strategy to study *tyrosinase *transgenes in mouse melanocytes

**DOI:** 10.1186/1471-2121-6-18

**Published:** 2005-04-12

**Authors:** Alfonso Lavado, Ander Matheu, Manuel Serrano, Lluís Montoliu

**Affiliations:** 1 Department of Molecular and Cellular Biology Centro Nacional de Biotecnología (CNB-CSIC) Campus de Cantoblanco, C/ Darwin, 3 28049 Madrid, Spain; 2 Spanish National Cancer Centre (CNIO) C/ Melchor Fernández Almagro 3 28029 Madrid, Spain; 3 St Jude Children's Research Hospital 332 N. Laudardale Memphis TN 38105, USA

## Abstract

**Background:**

A number of transgenic mice carrying different deletions in the Locus Control Region (LCR) of the mouse *tyrosinase (Tyr) *gene have been developed and analysed in our laboratory. We require melanocytes from these mice, to further study, at the cellular level, the effect of these deletions on the expression of the *Tyr *transgene, without potential interference with or from the endogenous *Tyr *alleles. It has been previously reported that it is possible to obtain and immortalise melanocyte cell cultures from postnatal mouse skin.

**Results:**

Here, we describe the efforts towards obtaining melanocyte cultures from our *Tyr *transgenic mice. We have bred our *Tyr *transgenic mice into *Tyr *^*c*-32*DSD *^mutant background, lacking the endogenous *Tyr *locus. In these conditions, we failed to obtain immortalised melanocytes. We decided to include the inactivation of the *Ink4a-Arf *locus to promote melanocyte immortalisation. For this purpose, we report the segregation of the *Ink4a-Arf *null allele from the *brown *(*Tyrp1*^*b*^) mutation in mice. Finally, we found that *Ink4a-Arf *^+/- ^and *Ink4a-Arf *^-/- ^melanocytes had undistinguishable tyrosine hydroxylase activities, although the latter showed reduced cellular pigmentation content.

**Conclusion:**

The simultaneous presence of precise genomic deletions that include the *tyrosinase *locus, such as the *Tyr *^*c*-32*DSD *^allele, the *Tyr *transgene itself and the inactivated *Ink4a-Arf *locus in *Tyrp1*^*B *^genetic background appear as the crucial combination to perform forthcoming experiments. We cannot exclude that *Ink4a-Arf *mutations could affect the melanin biosynthetic pathway. Therefore, subsequent experiments with melanocytes will have to be performed in a normalized genetic background regarding the *Ink4a-Arf *locus.

## Background

Eukaryotic genes are organised on chromosomes in units known as expression domains, that are believed to include all regulatory elements required for correct gene expression [1]. We use the mouse *tyrosinase *locus (*Tyr*) as an experimental model to study mammalian expression domains [2,3]. The mouse *Tyr *gene is located in chromosome 7 [4], encodes the rate-limiting enzyme in melanin biosynthesis and is tightly regulated during development, being exclusively expressed in neural crest-derived melanocytes and optic cup-derived retinal pigment epithelium (RPE) cells [5,6].

Classically, the approach used to functionally identify regulatory elements has been testing a series of DNA constructs containing different amounts of regulatory sequences in transgenic animals. Minigene *Tyr *constructs were able to rescue the albino phenotype of recipient animals, but displayed variability in pigmentation [7,8]. In contrast, the generation of transgenic mice with a 250 kb yeast artificial chromosome (YAC) covering the entire mouse *Tyr *locus completely rescued the albino phenotype, resulting in mice that were indistinguishable from agouti wild-type pigmented mice [9,10]. These results pointed to the existence of important regulatory elements, absent in previous standard constructs, such as the locus control region (LCR), identified 15 kb upstream of the mouse *Tyr *promoter [11,12]. The LCR is necessary to establish the proper expression pattern of the mouse tyrosinase gene. The absence of the LCR resulted in weaker pigmentation, variegated expression in the melanocytes and RPE cells and delayed retinal pigmentation in transgenic mice [13]. Moreover, two binding boxes for nuclear factors within the LCR core, known as boxes A and B, were identified by in vitro analysis [14] and, recently, have been incorporated into a new boundary activity associated within the LCR region [15]. We have generated transgenic mice with new YAC *Tyr *transgenes carrying a range of specific mutations within the LCR region [10].

To address a more detailed study, both at the functional and structural level (using biochemical and cellular approaches), of the role of LCR-variants in these different transgenes, a number of problems had to be solved, including: (1), the dispersed nature of melanocytes which prevented us from direct analysis of relevant tissues, such as skin, where many other unrelated cell types are found; (2), the *tyrosinase *albino allele (*Tyr *^*c*^), present in all recipient mouse strains used for the generation of transgenic mice, carrying a reported point-mutation within the coding region that results in a non-functional protein, without the transcriptional status of the locus being affected [16-18], and, [3], although it has been demonstrated that it is possible to obtain mouse melanocyte immortal cell lines from postnatal skins [19-22], often it becomes difficult to overcome the senescence period that all primary cell cultures undergo.

In this study we describe our efforts and the strategy to obtain melanocyte cell cultures from YAC *Tyr *transgenic mice in a genetic background lacking the endogenous mouse tyrosinase gene, and the effect of the inactivation of the *Ink4a-Arf *locus [23] on proliferation, senescence and tyrosinase activity of established melanocyte cell lines.

## Results and discussion

### Transfer of YAC *Tyr *transgenes from albino outbred NMRI mice (*Tyr *^*c *^/ *Tyr *^*c*^) to a *Tyr *^*c*-32*DSD *^/ *Tyr *^*c*-32*DSD *^background

**Figure 1:**
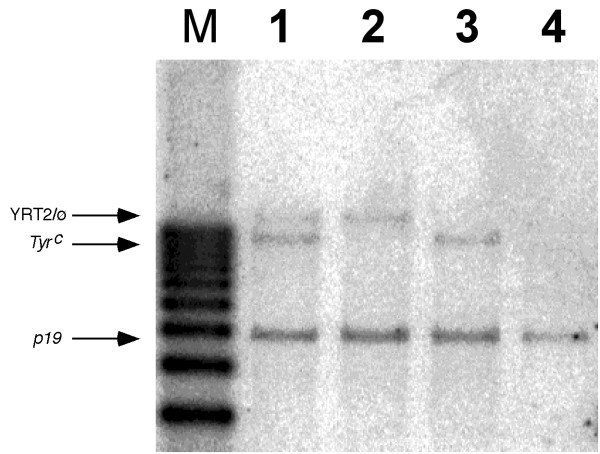
**Transfer of YAC *Tyr *transgenes from a *Tyr *^*c *^/ *Tyr *^*c *^background to a *Tyr *^*c*-32*DSD *^/ *Tyr *^*c*-32*DSD *^background. **Southern blot analysis of representative mice obtained during the breeding program, using the RFLP [9] and *p19*^*Arf *^E1 probes. Lanes: (M) 1 kb-ladder (Invitrogen), (1) YAC Tyr YRT2/∅ [9]; *Tyr *^*c*-32*DSD *^/ *Tyr *^*c*^, (2) YRT2/∅ ; *Tyr *^*c*-32*DSD *^/ *Tyr *^*c*-32*DSD*^, (3) ∅/∅ ; *Tyr *^*c*-32*DSD *^/ *Tyr *^*c*^, and (4) ∅/∅ ; *Tyr *^*c*-32*DSD *^/ *Tyr *^*c*-32*DSD*^. Note: ∅ indicates the absence of the transgene. Tg: YAC *Tyr *transgene derived signal. *Tyr *^*c*^: endogenous albino *tyrosinase *locus. p19: The p19Arf E1 probe (kindly provided by M. Malumbres), detecting exon 1 of the single-copy *p19arf *gene, was employed as an internal control for DNA loading and comparisons.

Previous *Tyr *transgenic mice have been generated in albino outbred NMRI mice [9,10,12]. All observed pigmentation is due to the expression of the *Tyr *transgene but the presence of mutant albino Tyr protein and mRNA from the host *Tyr *^*c *^allele [16-18] could interfere in subsequent cellular, biochemical and genomic analyses. The albino 32DSD mutant mouse (*Tyr *^*c*-32*DSD*^) carries a deletion of the entire *Tyr *locus, encompassing ~200 kb of mouse chromosome 7 [24]. Therefore, a breeding program was established between *Tyr *transgenic and albino 32DSD mice in order to obtain *Tyr *transgenes in animals lacking the endogenous *Tyr *alleles. Genotype analysis of the resulting mice was carried out by Southern blot. An *Eco*R I restriction fragment length polymorphism (RFLP) in exon 2 detected by the RFLP probe allows the identification of the transgenic *Tyr *allele (17 kb), derived from the mouse C3H strain, and the endogenous counterpart albino *Tyr *^*c *^NMRI allele (12 kb) (9) (Fig. 1).

### Melanocyte primary cultures of YAC *Tyr/∅ *; *Tyr *^*c*-32*DSD*^/ *Tyr *^*c*-32*DSD*^

To gain further insight on a series of YAC *Tyr *transgenic mice carrying a range of deletions around the LCR [10,12], we decided to prepare cell lines that could be representative of these animals. Chromatin analyses cannot be done in tissue samples obtained directly from transgenic animals, due to the low number of cells expressing the *Tyr *gene (RPE cells and melanocytes) and the complexity of the tissues or organs containing these cells (eye and skin, respectively). In addition, the presence of the mutated, but transcriptionally active [16-18], albino *Tyr *locus in all transgenic mice generated to date could interfere with the interpretation and the acquisition of experimental data. To avoid this problem we mobilised the YAC *Tyr *transgenes to a genetic background lacking the endogenous mouse gene, as shown in Fig. 1, and then we tried to establish melanocyte cultures from these mice.

**Figure 2:**
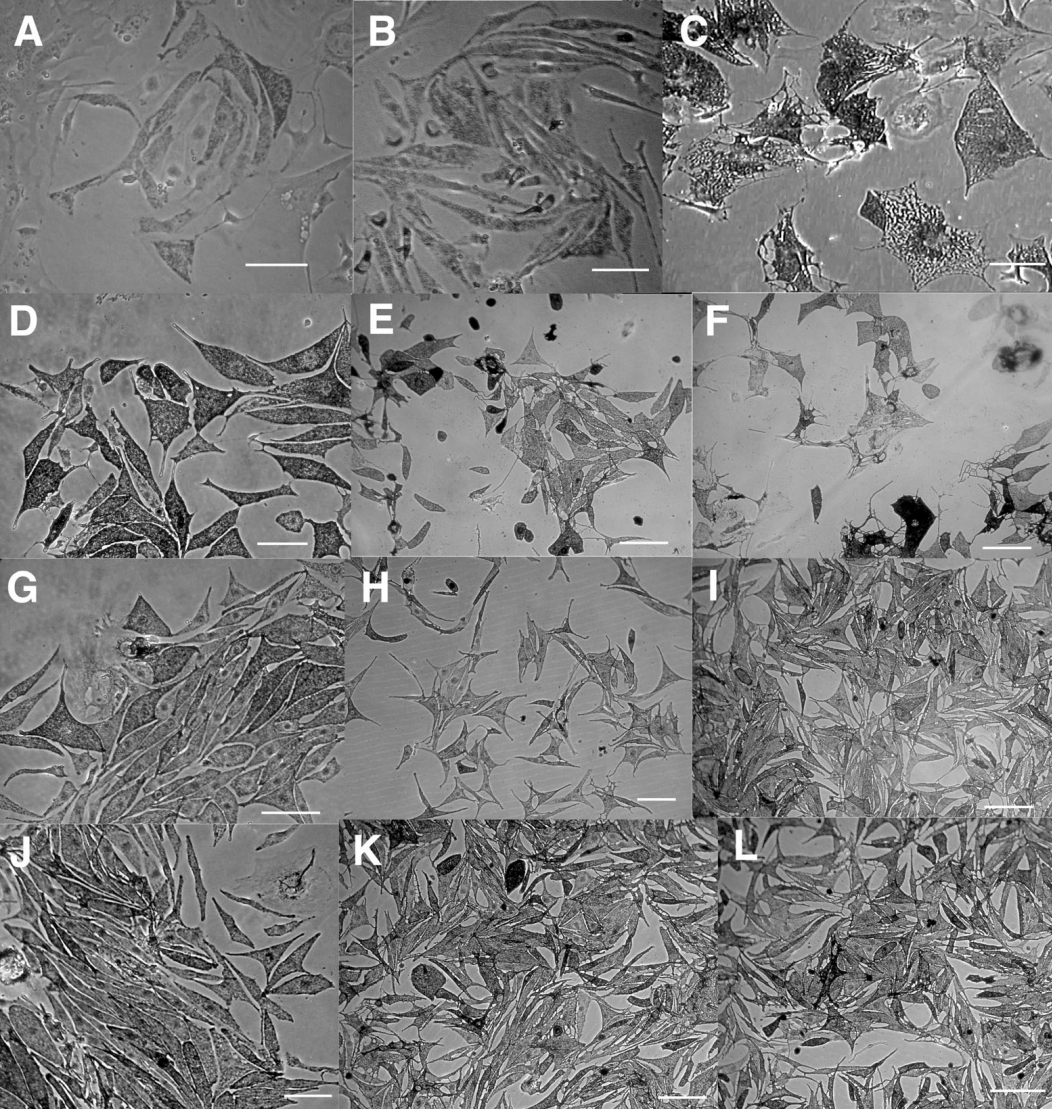
**The absence of at least one *Ink4a-Arf *allele overcomes the senescence of primary melanocytes in culture**. Melanocytes from heterozygous YAC *Tyr *transgenic in homozygous *Tyr *^*c*-32*DSD *^background became large, flat, vacuolated and highly pigmented (A, B). No surviving melanocytes were detected in the culture dishes after the senescence step (C). Melanocytes from *Ink4a-Arf *^+/- ^(G, H, I) and *Ink4a-Arf *^-/- ^(J, K, L) mutant mice in a *Tyrp1*^*B *^background are black, small, bipolar, pale and without significant sings of senescence, as compared to wild type *Ink4a-Arf *^+/+ ^melanocytes (D, E, F). A: culture at day 10; B: culture at day 18; C: culture at day 48; D, G, J: cultures at day 43; E, H, K: cultures at day 57; and, F, I, L: cultures at day 82. Scale ebars in C, D, G, J = 200 μm and in A, B, E, F, H, I, K, L = 150 μm.

Mouse melanocyte immortal cell lines can be derived from postnatal skins [19-22]. Mouse crosses were established with parental lines to obtain pigmented pups with the desired genotype (YAC *Tyr */ ∅ ; *Tyr *^*c*-32*DSD *^/ *Tyr *^*c*-32*DSD*^). Genotype analysis was not necessary to distinguish between transgenic and non transgenic pups, because melanin, clearly visible in the eye and in the skin at this stage, could only derive from the YAC *Tyr *transgene expression. Melanocyte primary cultures from dorsal skin of heterozygous YAC *Tyr *transgenes in homozygous *Tyr *^*c*-32*DSD *^background were prepared as described [19]. Individual melanocytes and small melanocytes colonies appeared at culture day 10. The number of cells increased slowly until day 25–35, when melanocytes showed signs of senescence. Most cells died by day 85–90 and no immortal cell lines could be established (Fig. 2A–C). Similar results were obtained from all YAC Tyr derivative transgenes bred to homozygous *Tyr *^*c*-32*DSD *^mice.

Mouse melanocyte primary cultures and their corresponding immortalised cell lines have been established from a number of mutant mice [25-29], although this type of cell lines can be sometimes difficult to achieve, as it may be inferred from these listed publications, in which reported cell lines have been generated by the same laboratory. A number of parameters can influence the success in obtaining immortalised melanocytes from mice. First, skins from mouse pups are used as starting material, carrying bacteria and other microorganisms that can contaminate the cultures. Second, and most important, melanocytes, as any somatic cell line in culture, undergo a senescence step previous to their immortalisation. Due to the low number or surviving cells after this senescence step, cells need to be cultured continuously during a minimum of 3–6 months to obtain an immortal cell line [19,20]. In most of the cultures we did not observe melanocytes after the senescence step (Fig. 2A–C). These results were obtained with all different primary cultures, regardless of their genotype, indicating a problem at the immortalisation step.

### Segregation of the *Ink4a-Arf *locus from the *Tyrp1 *^*b *^locus

It has been reported that melanocyte immortal cell lines (i.e. *melan-a *and *melan-c*) lack the p16 protein, most likely due to the lost of the *Ink4a-Arf *locus during the culture process [30]. Melanocytes from *Ink4a-Arf *(-/-) null mice proliferate exponentially without showing any signs of senescence, thus it has been proposed that the generation of melanocyte immortal cell lines in an *Ink4a-Arf *null background would be much easier [30]. Comparable results had been obtained before with fibroblast cultures from *Ink4a-Arf *homozygous mutant mice [31]. The absence of p16 leads to the inhibition in the inactivation of CDK4 and CDK6. These kinases inactivate the retinoblastoma pathway, promoting the proliferation of the cells [32,33]. Therefore, we decided to mobilise the *Ink4a-Arf *null allele into our YAC *Tyr *transgenic mice.

**Figure 3:**
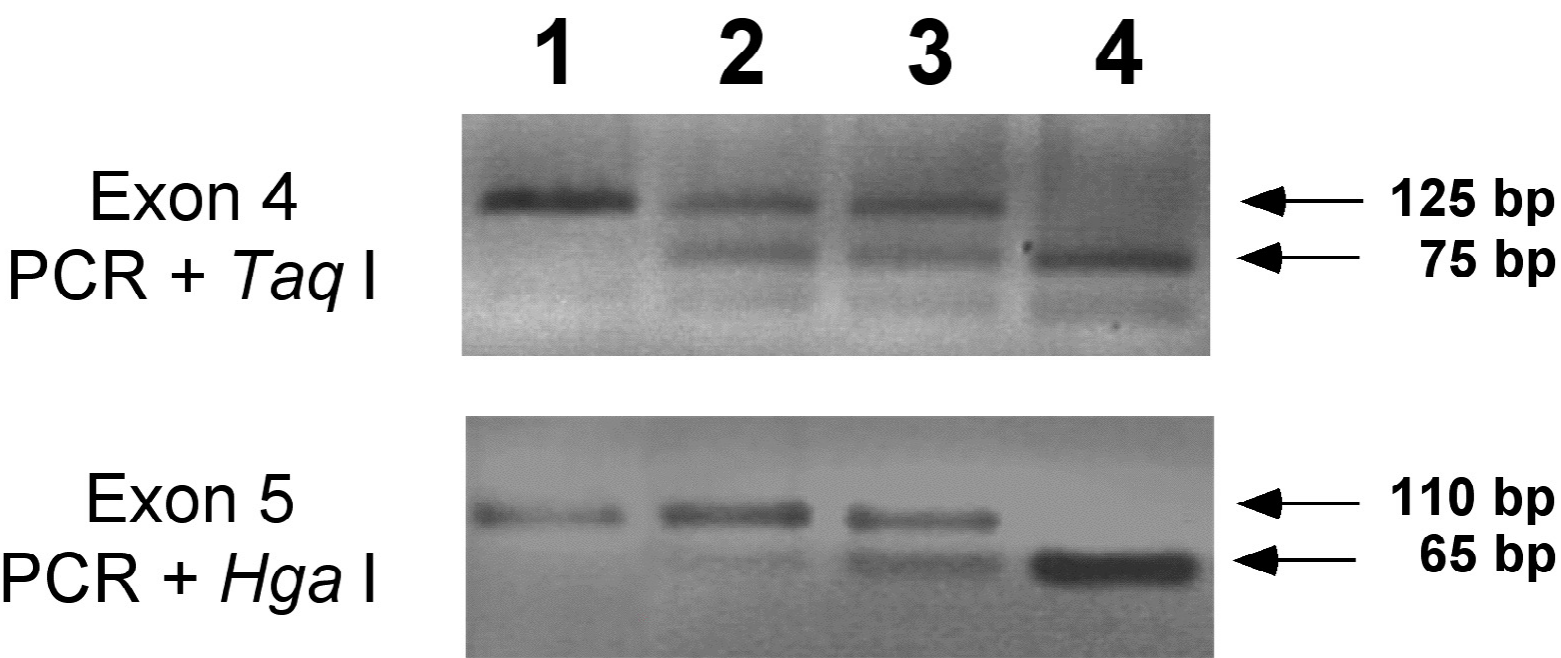
**Generation of an inbred *Ink4a/Arf *^-/-^; *Tyrp1 *^*B *^/ *Tyrp1 *^*B*^mouse strain**. Illustrative examples of the genotyping of the *Tyrp1 *locus by PCR and digestion with *Taq *I (A) or *Hga *I (B). Gel lanes correspond to: (1) *Ink4a/Arf *^-/- ^; *Tyrp1 *^*b *^/ *Tyrp1 *^*b*^, (2) *Ink4a/Arf *^+/- ^; *Tyrp1 *^*B *^/ *Tyrp1 *^*b*^, (3) *Ink4a/Arf *^-/- ^;*Tyrp1 *^*B *^/ *Tyrp1 *^*b *^and (4) *Ink4a/Arf *^-/- ^; *Tyrp1 *^*B *^/ *Tyrp1 *^*B*^.

First, we had to remove the *brown *mutation co-segregating with the *Ink4a-Arf *null allele. The murine *brown *locus corresponds to the gene encoding the Tyrosinase-related protein 1 (Tyrp1), an enzyme which is also implicated in the melanin biosynthetic pathway [34]. It was originally noted that the coat colour of *Ink4a-Arf *null mice was paler than their wild-type littermates [23]. Indeed, biochemical evidence was presented that was highly suggestive of defective Tyrp1 activity in *Ink4a-Arf *null melanocytes [35]. Homozygous *Tyrp1 *^*b *^mutant mice display less tyrosinase activity than wild type, because mutated forms of the Tyrp1 protein affect tyrosinase processing [36]. Notably, the *Tyrp1 *locus, is close to the *Ink4a-Arf *locus, in mouse chromosome 4, at a distance of 8.7 Mb (mouse ENSMBL, build 32). Most inbred laboratory strains with defective Tyrp1 activity carry the recessive *brown *allele, *Tyrp1 *^*b *^that contains a single amino acid change at a critical residue in the Tyrp1 protein [37]. To test directly whether the *Ink4a-Arf *null allele was linked to the *Tyrp1 *^*b *^allele, two single nucleotide polymorphisms (SNPs) that characterize the *Tyrp1 *^*b *^allele and that can be diagnosed by subsequent restriction enzyme digestion [37] were used. In particular, a *Tyrp1 *^*b*^-linked SNP at exon 4 eliminates a *Taq *I restriction site and, similarly, another *Tyrp1 *^*b *^– linked SNP at exon 5 eliminates an *Hga *I restriction site. Using these two markers, we directly proved that the original *Ink4a-Arf *null mice were indeed homozygous for the *Tyrp1 *^*b *^allele, thus explaining their paler coat colour (Fig. 3). The *Tyrp1 *^*b *^recessive allele linked to the mutated *Ink4a-Arf *allele was probably derived from the ES cell line originally used for targeting the *Ink4a-Arf *locus, namely WW6 ES cells, which had a complex genetic background [38].

*Ink4a/Arf-*null strain in a pure C57BL/6J genetic background and unlinked from the mutant *Tyrp1 *^*b *^allele were obtained to avoid the confounding presence of the *Tyrp1 *^*b *^allele. *Ink4a-Arf *^+/- ^mice were backcrossed seven times with wild-type C57BL/6J mice, eventually yielding *Ink4a-Arf *^+/- ^; *Tyrp1 *^*B*^/ *Tyrp1 *^*b *^mice. These mice were intercrossed to produce a number of *Ink4a-Arf *^-/- ^mice that were in their majority *Ink4a-Arf *^-/- ^; *Tyrp1 *^*b*^/ *Tyrp1 *^*b *^and, accordingly, had a brown coat. Exceptionally, one mouse (from a total population of 27 *Ink4a-Arf *^-/- ^mice) was identified as an *Ink4a-Arf *^-/- ^but had a black coat. This mouse turned out to represent a recombinant with an *Ink4a-Arf *^-/- ^; *Tyrp1 *^*B*^/ *Tyrp1 *^*b *^genotype. From this animal, and after the appropriate crosses, a strain of mice in C57BL/6J background that were *Ink4a-Arf *^-/- ^; *Tyrp1 *^*B *^/ *Tyrp1 *^*B *^was obtained (Fig. 3), and used for subsequent experiments.

### Melanocyte primary cultures from *Tyrp1 *^*B*^/ *Tyrp1 *^*B *^*Ink4a-Arf *mutant mice

**Figure 4:**
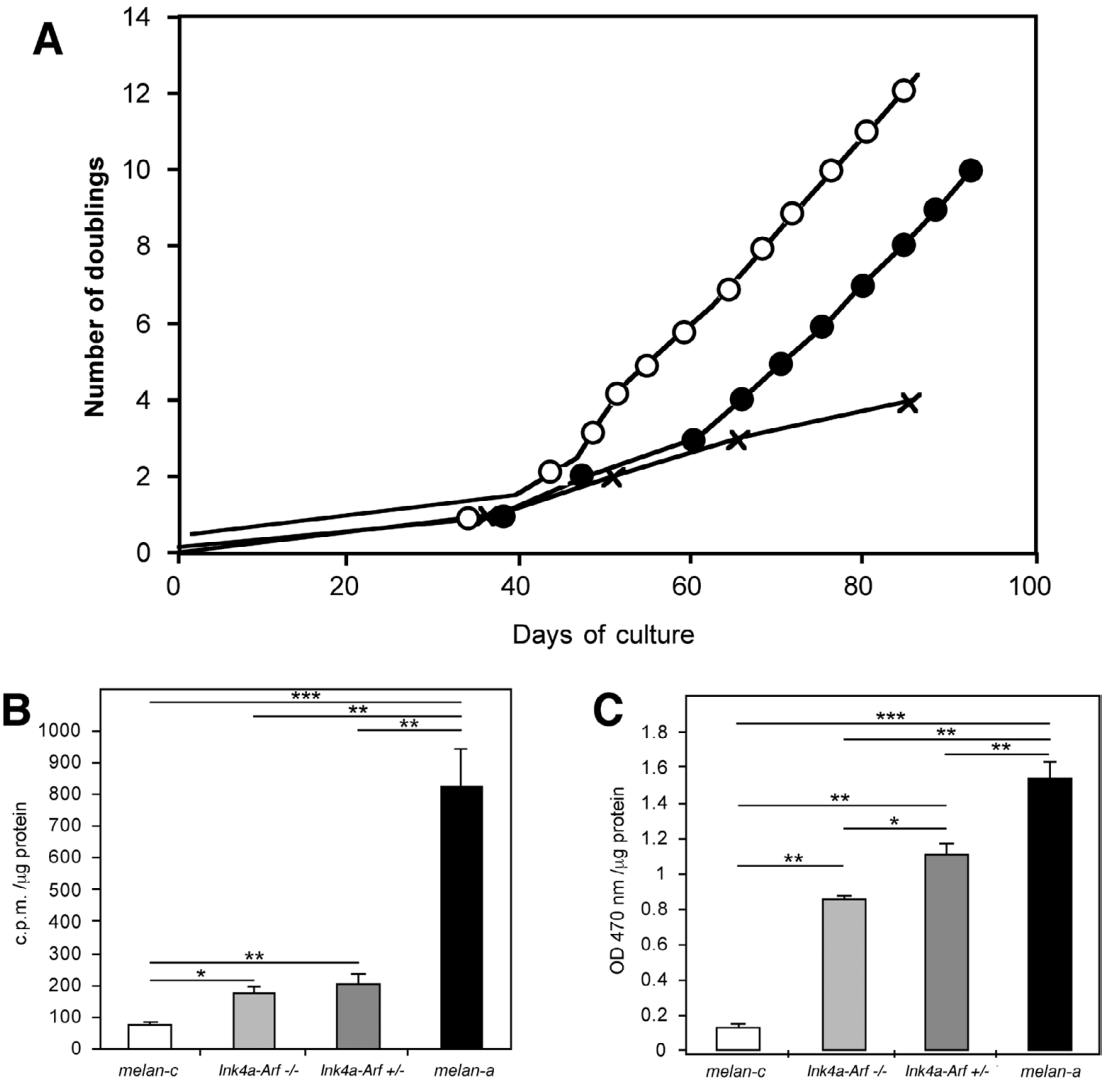
**The effect of *Ink4a-Arf *locus on cell growth and *Tyr *enzymatic activity. **(A) Cell growth of *Ink4a-Arf *^-/- ^(open circles), *Ink4a-Arf *^+/- ^(closed circles) and *Ink4a-Arf *^+/+ ^(crosses) melanocytes. Each point represents one subculture step and represents the mean count of cells from three replicates. (B) Tyrosine hydroxylase activity in albino *melan-c *(white bars), [*Ink4a-Arf *^+/-^; *Tyrp1 *^*B *^/ *Tyrp1 *^*B *^] (dark-grey bars), [*Ink4a-Arf *^-/- ^; *Tyrp1 *^*B *^/ *Tyrp1 *^*B *^] (light-grey bars) and *melan-a *(black bars) cells. (C) Melanin content in the same cellular types, as in (B). Bars represent the mean value (+/- SD) from three replicates. Tyrosinase activity and melanin content were measured in melanocyte primary cultures at passage 8 (~day 70 of culture). Statistically significant differences (*t*-Student test) are indicated as follows: * p < 0,1; ** p < 0,01; *** p < 0,001.

To evaluate the effect of the inactivation of the *Ink4a-Arf *locus in *Tyrp1 *^*B *^/ *Tyrp1 *^*B *^genetic background on the immortalisation of mouse melanocytes, melanocyte primary cultures from dorsal skin of *Ink4a-Arf *^+/+^, *Ink4a-Arf *^+/-^, *Ink4a-Arf *^-/- ^pups in a *Tyrp1 *^*B*^background were prepared (Fig. 2D–L), using the same described procedures [19]. Individual melanocytes and colonies appeared at day 10. The number of cells increased slowly in *Ink4a-Arf *^+/+ ^(Fig. 4A) and, around day 35, melanocytes increased their size and lost their bipolar shape. Few or none *Ink4a-Arf *^+/+ ^melanocytes survived the senescence step, and most of these melanocytes died by day 85–90 (Fig. 2D–F). In contrast, *Ink4a-Arf *^+/- ^and *Ink4a-Arf *^-/- ^kept their bipolar shape and small size, with a limited number of cells showing any sign of senescence (Fig. 2G–L). Proliferation of *Ink4a-Arf *^-/- ^melanocytes was higher than in *Ink4a-Arf *^+/- ^melanocytes, and both were much higher than in *Ink4a-ARF *^+/+ ^melanocytes (Fig. 4A). By day 45 of culture, *Ink4a-Arf *^-/- ^melanocytes entered the exponential phase of growth. In contrast, *Ink4a-Arf *^+/- ^melanocytes did not show evidences of exponential growth until day 65 (Fig. 4A). This delay could be explained by LOH (loss of heterozygosity), spontaneously occurring in *Ink4a-Arf *^+/- ^melanocytes and affecting the remaining wild-type allele of the *Ink4a-Arf *locus, a common event that is known to take place both *in vitro *[39] and *in vivo *[40]. Similar results have been reported [30] using independent mouse colonies.

### Tyrosinase activity and melanin content in the melanocyte cultures from *Ink4a-Arf *mutant mice

A valid approach to analyse the role of the different regulatory regions of the mouse *Tyr *gene in the transcription of the locus and, eventually, in the amount of mature protein being made, is the measurement of the enzymatic activity of the derived Tyr protein. We measured the levels of Tyr enzymatic activity and melanin content using reported procedures [41] in melanocyte cultures, to study the influence of the presence or absence of the p16 protein in the expression of the *Tyr *gene. Tyrosine hydroxylase activity values from *Ink4a-Arf *^-/- ^and *Ink4a-Arf *^+/- ^cell extracts were undistinguishable and significantly lower to values obtained in *melan-a *cells (Fig. 4B). However, the quantity of melanin in the proliferative *Ink4a-Arf *^-/- ^melanocytes cell cultures was significantly lower than in *Ink4a-Arf *^+/- ^or *melan-a *cells (Fig. 4C).

Differences in melanin content could be explained by different individual cell culture response to TPA or/and CT that are present in cell culture medium. However, the observed differences in cellular pigmentation were maintained after removing CT from cell culture medium. In addition, TPA is always required as an additive to maintain cellular proliferation. Increasing the amount of TPA does not result in a parallel increase in pigmentation, opposite to what is observed with CT.

Differences in melanin content could also be explained by the control of the retinoblastoma (RB) protein by p16, and the observation that RB protein interacts with the transcription factor microphthalmia (Mift) [42], that controls the expression of the *Tyr *gene. Differences between *Ink4a-Arf *mutant cells and *melan-a *could be due to the presence of a number of additional alterations in this latter immortal cell line, such as the loss of expression of *p16Ink4a *[30]. The recent generation of mice with increased gene dosage of *Ink4a-Arf *will be instrumental to further investigate the influence of this locus on Tyr activity [43].

## Conclusion

With all these results we can conclude that the simultaneous presence of: [1] at least one mutant allele of the *Ink4a-Arf *locus; [2], the *Tyr *^*c*-32*DSD *^mutant albino allele in homozygosis and; [3], the presence of the relevant *Tyr *transgene in heterozygosis, are required for the establishment and the study of immortal mouse melanocyte cultures from transgenic mice carrying *Tyr *constructs. Finally, we cannot exclude that *Ink4a-Arf *mutations could affect the melanin biosynthetic pathway. Therefore, experiments with mouse melanocytes will have to be performed in a normalized genetic background regarding the *Ink4a-Arf *locus.

## Methods

### Animals

Four types of mice were used in this study: YRT2 YAC *tyrosinase *heterozygous transgenic mice in an albino outbred NMRI background (YRT2/∅ ; *Tyr *^*c *^/ *Tyr *^*c*^) (line #1999) [9], 32DSD radiation induced albino mutant mice (*Tyr *^*c*-32*DSD*^/ *Tyr *^*c*-32*DSD*^) [24], homozygous *Ink4a-Arf *mutant mice (*Tyr *^+ ^/ *Tyr *^+ ^; *Tyrp1 *^*b *^/ *Tyrp1 *^*b *^; *Ink4a-Arf *^-/-^) in C57BL/6J genetic background [23] and wild-type pigmented C57BL/6J mice. All experiments complied with local and European legislation concerning vivisection and the experimentation and use of animals for research purposes.

### Southern blot analysis

The discrimination of the endogenous *tyrosinase *gene from the YAC-*tyrosinase *transgenes was performed as previously described, using the RFLP probe, containing exon 2 of the mouse *tyrosinase *gene [9,12]. To obtain an endogenous internal control, membranes were co-hybridised with a single-copy mouse gene, the *p19*^*Arf *^E1 probe, a *Eco*R I DNA fragment, 230 bp in length, containing exon 1 of the *p19*^*Arf *^gene (pRSp19arfE1 plasmid, generous gift from M. Malumbres). In brief, genomic DNA was isolated from mice tail tips and prepared for *southern blot *as described [12]. 15–20 μg of genomic DNA were digested with *Eco*R I (Roche, Basel, Switzerland), fractionated by horizontal gel electrophoresis in 0,8% agarose and transferred to a Hybond-N nylon membrane (Amersham, Buckinghamshire, UK) by capillary blotting. RFLP and *p19*^*Arf *^E1 DNA probes were labelled with [α^32^P] dCTP using the High Prime labelling kit (Roche). Membranes were hybridised in Southern hybridisation solution (0.25 M Na_2_HPO_4 _pH = 7.2, 7% SDS, 1% BSA) overnight at 65°C, washed at 65°C in 20 mM Na_2_HPO_4 _pH = 7.2, 1% SDS, 1 mM EDTA pH = 8 and resulting blots exposed for 1–3 days and scanned with a PhosphorImager (Molecular Dynamics, Sunnyvale, CA, USA). Quantification of the hybridisation signals was performed using the ImageQuant v1.2 software (Molecular Dynamics).

### Genotyping of the *Ink4a-Arf *and *Tyrp1 *loci

The *Ink4a-Arf *and *Tyrp1 *loci were genotyped by PCR as follows. The *Ink4a-Arf *wild-type allele was specifically detected using primers that amplify *Ink4a-Arf *exon 2: mp16F, 5'-ATGATGATGGGCAACGTTC-3' and mp16R, 5'-CAAATATCGCACGATGTC-3'. The *Ink4a-Arf *null allele, which has a *neo*-cassette substituting exons 2 and 3 [23], was genotyped using primers that hybridise, respectively, with the *neo*-cassette (oligo Neo) and with *Ink4a-Arf *flanking genomic sequences (oligo R1): Neo, 5'-CTATCAGGACATAGCGTTGG-3' and R1, 5'-AGTGAGAGTTTGGGGACA GAG-3'. To genotype the *Tyrp1 *locus, two different PCR reactions were performed to amplify, respectively, exons 4 and 5 of the *Tyrp1 *gene. Amplification of exon 4 was performed using primers: Tyrp1-4F, 5'-CTGCGATGTCTGCACTGATGACTT-3' and Tyrp1-4R, 5'-AGGGTATCGTACTCTTCCAAGGAT-3'. Amplification of exon 5 was performed using primers: Tyrp1-5F, 5'-ACAGCACTGAGGGTGGACCAATC-3' and Tyrp1-5R, 5'-AGGGTATCGTACTCTTCCAAGGAT-3'. After amplification, the product corresponding to exon 4 was digested with *Taq *I, and the product corresponding to exon 5 was digested with *Hga *I. Digestion mixtures were separated in standard agarose gels and visualized with ethidium bromide. The *Tyrp1 *^*b*^mutant allele does not contain neither of the previous restriction sites, *Taq *I and *Hga *I, whereas both enzymes digest the *Tyrp1 *^*B *^wild-type allele. PCR reactions had 3 mM MgCl_2_, 1% DMSO, 2.5 mM dNTPs (Epicentre, Madison, WI, USA), 20 pmol of each primer, and 0.25 μl of Taq-Gold (Applied Biosystems, Foster City, CA, USA). In addition, each reaction had approximately 100 ng of genomic DNA extracted from the tail tips. Annealing temperature was 60°C and PCR reactions were carried on for 30 amplification cycles.

### Melanocyte cultures

Melanocyte cultures were prepared, essentially, as previously described [19]. Briefly, dorsal skin biopsies were obtained from pups of all investigated genotypes between +19.5 and +22.5 d.p.c. stages (postnatal P2–P3). Dorsal skin was split in 5 μg/ml trypsin (Sigma, St. Louis, MO, USA) in PBS and the epidermal layer then minced with a pair of surgical blades in 250 μg/ml trypsin and 200 μg/ml EDTA in PBS. Cells were cultured on a feeder layer of mitomycin-treated murine XB2 keratinocytes [44]. The cells were grown in RPMI-1640 medium containing 2 mM glutamine, 10% fetal calf serum, 100000 U/l penicillin, 100 mg/l streptomycin sulphate (all from Invitrogen, Carlsbad, CA, USA), 200 nM tetradecanoyl phorbol acetate (TPA) (Sigma) and 200 pM cholera toxin (CT) (Sigma), at 37°C, 95% humidity and 10% CO_2 _pressure. Explant cultures from different donor mice were kept separate. Passages were made when cultures became subconfluent, and melanocytes were counted at each passage. Feeder cells were added when necessary. Control *melan-a *and *melan-c *cells, derived from inbred C57BL/6J and outbred albino LAC-MF1 mice, respectively, were cultured as previously described [19,20].

### Quantification of melanin and tyrosinase enzymatic activities

Melanin contents in whole cell extracts were measured by spectrophotometer essentially as described [45]. In brief, 6 × 10^6 ^cells from ~day 70 of culture were collected and homogenised in 300 μl of PBS, 100 μl of homogenate incubated for 14–16 hours at room temperature with 900 μl of 2 M NaOH, 20% DMSO and absorbance measured at 470 nm.

Tyrosinase enzymatic activities were recorded following described assays [41,46,47]. In brief, for tyrosine hydroxylase activity, 6 × 10^6 ^cells from ~day 70 of culture were collected and cell extracts were prepared in 10 mM Sodium Phosphate buffer pH = 6.8 to which Tween-20 (Igepal) was added (1% final concentration) prior the assay. Reaction volume included 10 μl of L-DOPA 250 mM, 10 μl of L-[3,5-^3^H]-Tyrosine mix (450 μl of L-Tyrosine 262 μM in 10 mM Sodium Phosphate buffer pH = 6.8 and 50 μl L-[3,5-^3^H]-Tyrosine [1 mCi/ml, 46 Ci/mmol, Amersham]), 20 μl of Sodium Phosphate buffer pH = 6.8 and 10 μl of cell extracts, was incubated for 1 hour at 37°C, and stopped by adding 450 μl trichloracetic acid (TCA) 1% (Merck, Darmstadt, Germany). A small amount of absorbing substrate (active carbon [Merck] and Celite 545 [Fluka, St. Gallen, Switzerland], 1:1) was added, mixed for 30 min at room temperature and centrifuged. Radioactivity from clear supernatants (100 μl) was measured in a β-scintillation counter (Beckmann, Fullerton, CA, USA).

## Authors' contributions

AL carried out the molecular genetic, cellular, biochemical and statistical studies and drafted the manuscript, MS conceived of the segregation of *ink4a-Arf *from mutant *brown *locus, AM carried out the genotype analysis of *ink4a-Arf *mice. LM conceived of the general study, and participated in its design and coordination, in collaboration with MS, and helped to draft the manuscript. MS also helped to draft the manuscript. All authors read and approved the final manuscript.
